# Metabolic Dysfunction in Psychiatric Disorders: A Narrative Review of Shared Pathways Between Mental Illness and Cardiometabolic Disease

**DOI:** 10.7759/cureus.111469

**Published:** 2026-06-25

**Authors:** Pooja Mohanty, Mohsin Uddin, Kumar Sambhav, Sruthi S, Niraj Lodha, Abhishek Anand, Shraddha Basu

**Affiliations:** 1 Department of Psychotherapy and Counselling, Xavier School of Management (XLRI), Jamshedpur, Jamshedpur, IND; 2 School of Excellence in Panchayati Raj, National institute of Rural Development and Panchayati Raj, Hyderabad, IND; 3 Department of Anatomy, All India Institute of Medical Sciences, Bilaspur, Bilaspur, IND; 4 Department of Pharmacology, Sree Balaji Medical College and Hospital, Chennai, IND; 5 Department of Internal Medicine, Lodha Hospital and Research Centre, Pali, IND; 6 Department of Pharmacy Practice, Teerthanker Mahaveer College of Pharmacy, Teerthanker Mahaveer University, Moradabad, IND; 7 Department of Psychology, The Assam Royal Global University, Guwahati, IND

**Keywords:** cardiometabolic disease, inflammation, insulin resistance, psychiatric disorders, psychotropic medications

## Abstract

Psychiatric disorders and cardiometabolic disease (CMD) frequently coexist and contribute to substantial morbidity, premature mortality, and reduced quality of life. Although this association is increasingly recognized, the shared biological and clinical pathways linking mental illness with metabolic dysfunction remain incompletely defined. This article is a narrative review that examines recent evidence on mechanisms connecting depression, anxiety, schizophrenia, severe mental illness (SMI), obesity, insulin resistance, diabetes, dyslipidemia, hypertension, and cardiovascular disease. A narrative review approach was used, with literature identified from major biomedical databases and prioritized according to relevance to shared psychiatric-metabolic mechanisms, clinical significance, recency, and methodological strength. The review focuses on chronic low-grade inflammation, hypothalamic-pituitary-adrenal (HPA) axis dysregulation, insulin resistance, oxidative stress, mitochondrial dysfunction, altered brain energy metabolism, gut microbiome disruption, sleep and circadian disturbance, psychotropic medication effects, and behavioral and social determinants.

Current evidence suggests that psychiatric and cardiometabolic disorders are associated through bidirectional and overlapping pathways rather than a single causal mechanism. Metabolic dysfunction may be associated with worsening psychiatric symptoms through inflammatory, neuroendocrine, and neurotrophic effects, while psychiatric illness may increase cardiometabolic risk (CMR) through stress biology, lifestyle factors, medication exposure, and fragmented healthcare access. Antipsychotic-associated weight gain and glucose-lipid abnormalities remain important, but metabolic changes may also precede long-term treatment exposure, particularly in psychotic disorders. These findings support routine metabolic screening, early risk stratification, individualized psychotropic prescribing, lifestyle intervention, and integrated care involving psychiatry, primary care, endocrinology, cardiology, nutrition, and behavioral health services. Future longitudinal studies and randomized trials are needed to clarify causality and evaluate interventions that improve both psychiatric and cardiometabolic outcomes. Metabolic health should be considered a core component of psychiatric assessment and management.

## Introduction and background

The development of metabolic dysfunction has emerged as a significant area of concern in psychiatric medicine, as abnormalities in adiposity, glucose regulation, lipid metabolism, blood pressure, and systemic inflammation frequently co-occur with psychiatric illness. Major depressive disorder, anxiety disorders, bipolar disorder, schizophrenia, and stress-related illnesses are increasingly understood as conditions with systemic correlates involving immune, endocrine, metabolic, and behavioral pathways. This interpretation should be viewed as an association-based framework rather than evidence of a single causal pathway. For clarity, cardiometabolic disease (CMD) refers to major metabolic and cardiovascular conditions such as obesity, insulin resistance, diabetes, dyslipidemia, hypertension, and cardiovascular disease, while cardiometabolic risk (CMR) refers to the combined risk burden created by these abnormalities. The introduction first outlines the epidemiological and public health relevance of psychiatric-metabolic comorbidity and then describes biological, behavioral, treatment-related, and structural mechanisms that may explain why these disorders frequently overlap. A cross-sectional analysis of inflammatory and metabolic dysregulation reported that inflammatory and metabolic profiles were associated with different depressive symptom patterns, suggesting the possibility of clinically relevant metabolic-inflammatory depressive subtypes [1]. A study examining inflammatory markers in relation to depression and anxiety reported associations between inflammation and affective symptoms, supporting the concept that mood and anxiety symptoms may occur alongside systemic immune activation rather than being restricted to central nervous system processes alone [2]. Cohort evidence suggests that inflammatory cytokine profiles may be temporally associated with depressive outcomes, indicating a possible longitudinal relationship between inflammatory activity and psychiatric symptom burden without establishing causality [3]. A longitudinal biomarker study also reported complex bidirectional associations between inflammatory biomarkers and depression, supporting the need to consider psychiatric illness within a broader biological and clinical context [4].

A clinically relevant association is seen in the high cardiometabolic burden reported among individuals with psychiatric disorders. Obesity, dyslipidemia, insulin resistance, type 2 diabetes, hypertension, and cardiovascular disease are more common in people with severe mental illness (SMI), including schizophrenia and related psychotic disorders. A meta-review of meta-analyses of randomized controlled trials found that pharmacological and non-pharmacological interventions may improve physical health outcomes in people with schizophrenia, indicating that CMR may be clinically modifiable in this population [5]. A nationwide cohort study of adults with new-onset type 1 diabetes found that comorbid mental disorders were associated with all-cause mortality and cardiovascular disease outcomes, suggesting that psychiatric comorbidity may identify a higher-risk subgroup within diabetes populations [6]. A large population-based study reported broad associations between mental disorders and subsequent medical conditions, supporting a reciprocal clinical relationship between mental and physical health rather than a unidirectional causal model [7]. A meta-analysis focused on developing and emerging countries found that the co-occurrence of mental disorders and chronic physical diseases represents a significant public health concern across diverse settings [8]. These epidemiological findings are important because they show that psychiatric-metabolic comorbidity is not confined to specialist psychiatric populations; it is a broader clinical and public health problem involving premature mortality, preventable physical disease, and unequal access to preventive care.

Behavioral and social determinants may further modify this association. Individuals with psychotic disorders often have lower physical activity, poorer diet quality, sleep disturbance, smoking history, substance use, chronic healthcare barriers, and limited access to preventive care. A two-year follow-up study of an individualized lifestyle intervention reported improvements in health-related outcomes among people with psychotic disorders receiving psychiatric outpatient care, suggesting that sustained and integrated lifestyle support may be beneficial [9]. Vulnerability is also apparent in nonpsychotic populations with cardiovascular disease. A cohort study of veterans reported higher CMR among individuals with mental illness, suggesting that cardiometabolic vulnerability extends across different psychiatric subgroups and clinical settings [10]. Taken together, these studies support a multifactorial association in which biological, behavioral, pharmacological, and structural factors may interact with cardiometabolic dysfunction in psychiatric populations, rather than demonstrating a single causal pathway.

Another aspect of this relationship is treatment-related metabolic risk. Schizophrenia is commonly treated with antipsychotic drugs that play a critical role in symptom control and relapse prevention, but some of these medications are associated with weight gain, insulin resistance, and dyslipidemia, requiring structured cardiometabolic monitoring. A nationwide cohort study reported associations between antipsychotic exposure and mortality outcomes in schizophrenia, highlighting the need to balance psychiatric stability with long-term physical health considerations [11]. The under-recognition of cardiometabolic prevention may have important clinical implications. A systematic review and meta-analysis found reduced life expectancy and years of potential life lost among individuals with schizophrenia, with excess mortality substantially related to physical disease burden [12]. A systematic review of secure psychiatric settings also found that individuals with psychotic disorders had a high prevalence of CMD, supporting the need for population-specific screening and prevention strategies in institutionalized and forensic psychiatric populations [13].

**Figure 1:**
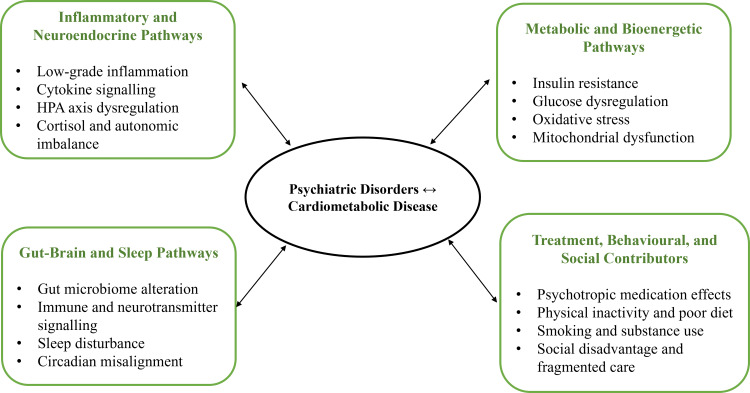
Shared Biological and Clinical Pathways Linking Psychiatric Disorders and Cardiometabolic Disease. Created by the authors using Microsoft PowerPoint (Microsoft Corp., Redmond, WA). This figure illustrates a bidirectional psychiatric-metabolic model in which inflammatory, neuroendocrine, metabolic, bioenergetic, gut-brain, sleep-related, treatment-related, behavioral, and social pathways may link psychiatric disorders with cardiometabolic disease. The pathways may contribute to clinical outcomes, including psychiatric symptom burden, cognitive dysfunction, metabolic syndrome, cardiovascular disease, premature mortality, and reduced quality of life. Arrows indicate overlapping and bidirectional associations rather than confirmed causal pathways. HPA: hypothalamic-pituitary-adrenal

These findings support an integrated model of psychiatric care with greater attention to physical health. A consensus and policy-oriented review stated that improving physical health among individuals with mental health conditions should be a fundamental part of psychiatric care rather than an add-on service [14]. A mechanistic narrative review described metabolic syndrome in psychiatric patients as being associated with inflammatory, neuroendocrine, autonomic, oxidative, mitochondrial, medication-related, and lifestyle pathways [15]. Because much of the evidence is observational, heterogeneous, or mechanistic, these pathways should be interpreted as overlapping associations and biologically plausible mechanisms rather than definitive causal explanations. Several unresolved questions justify this review: whether cardiometabolic dysfunction is primarily driven by psychiatric illness, psychotropic treatment, social and behavioral risk, preexisting metabolic disease, or interaction among these factors; whether these pathways differ across diagnoses; and which biomarkers or interventions have sufficient clinical utility for routine practice. Therefore, this review explores shared biological and clinical pathways between psychiatric illnesses and cardiometabolic conditions, including inflammation, dysregulated stress biology, insulin resistance, mitochondrial dysfunction, gut-brain-metabolic signalling, sleep and circadian disturbance, effects of psychotropic medications, screening, prevention, and integrated care. Figure 1 provides a conceptual system map of these bidirectional psychiatric-metabolic associations, showing how biological, treatment-related, behavioral, and social pathways may converge on psychiatric symptom burden, metabolic syndrome, cardiovascular disease, reduced quality of life, and premature mortality.

Objectives of the review

The objective of this narrative review is to explore the association between psychiatric disorders and cardiometabolic disease (CMD) by summarizing shared biological and clinical pathways, including chronic inflammation, hypothalamic-pituitary-adrenal (HPA) axis dysregulation, insulin resistance and glucose dysregulation, oxidative stress, mitochondrial dysfunction, gut-brain-metabolic axis alterations, sleep and circadian disturbance, psychotropic medication effects, and behavioral and social determinants. For consistency, SMI refers to disabling psychiatric conditions such as schizophrenia, bipolar disorder, and related severe psychiatric disorders; CMD refers to obesity, insulin resistance, diabetes, dyslipidemia, hypertension, and cardiovascular disease; and cardiometabolic risk (CMR) refers to the combined risk burden arising from these metabolic and cardiovascular abnormalities. This review also assesses the role of psychotropic medications and lifestyle-related factors in metabolic vulnerability and identifies implications for screening, prevention, integrated care, and future research. Because this is a narrative review, the objective is to provide a clinically oriented synthesis of selected mechanistic, epidemiological, and interventional evidence rather than a quantitative or systematic evidence estimate.

Methodology

Review Design

This article was designed as a narrative review rather than a systematic review or meta-analysis. The aim was to synthesize current evidence on shared biological and clinical pathways linking psychiatric disorders with CMD, with emphasis on mechanisms relevant to clinical assessment, prevention, and integrated care. Because this was a narrative review, the search was intended to identify clinically and mechanistically relevant literature rather than to provide an exhaustive systematic evidence map.

Literature Search Strategy

Literature was identified through searches of PubMed/Medical Literature Analysis and Retrieval System Online (MEDLINE), Google Scholar, Scopus, and Web of Science. Search terms included combinations of “psychiatric disorders,” “severe mental illness,” “depression,” “anxiety,” “schizophrenia,” “cardiometabolic disease,” “metabolic syndrome,” “insulin resistance,” “diabetes,” “obesity,” “dyslipidaemia,” “hypertension,” “cardiovascular disease,” “inflammation,” “HPA axis,” “oxidative stress,” “mitochondrial dysfunction,” “gut microbiome,” “sleep disturbance,” “circadian rhythm,” “psychotropic medications,” and “antipsychotic-induced weight gain.”

Eligibility Criteria

The search prioritized English-language peer-reviewed articles published mainly from 2016 to 2026, while older foundational articles were included when they provided important mechanistic or clinical context. Eligible sources included systematic reviews, meta-analyses, cohort studies, randomized or interventional studies, consensus statements, and clinically relevant narrative reviews addressing psychiatric-metabolic overlap, shared biological mechanisms, treatment-related metabolic risk, sleep and circadian disturbance, or integrated care. Articles were excluded if they were not directly related to psychiatric disorders and cardiometabolic outcomes, lacked relevance to the review objectives, focused only on unrelated neurological or endocrine conditions, or were not available in English.

Study Selection and Narrative Synthesis

Study selection was conducted by screening titles and abstracts for relevance, followed by a full-text review of articles considered central to the topic. Evidence was synthesized narratively and organized by major mechanistic and clinical themes: chronic inflammation, HPA axis and stress biology, insulin resistance and glucose metabolism, oxidative stress and mitochondrial dysfunction, gut-brain-metabolic signalling, sleep and circadian disturbance, psychotropic medication effects, lifestyle factors, screening, prevention, and integrated care. Greater weight was given to recent systematic reviews, meta-analyses, large cohort studies, and studies with direct clinical implications.

Risk-of-Bias and Statistical Analysis

Although formal risk-of-bias scoring was not performed, included studies were critically appraised narratively according to study design, sample size, population relevance, the clarity of outcome definitions, methodological transparency, recency, and applicability to psychiatric-metabolic clinical care. Because this was a narrative review rather than a systematic review, formal risk-of-bias scoring and quantitative evidence grading were not performed; however, the methodological strengths and limitations of key evidence were considered during synthesis and are reflected in the interpretation of findings. Preferred Reporting Items for Systematic Reviews and Meta-Analyses (PRISMA) flow reporting and formal study-level risk-of-bias scoring were not applied because this review was not designed as a systematic review; however, PRISMA-style transparency principles were used to describe the search sources, eligibility criteria, study selection, and synthesis approach. Because no quantitative synthesis was performed, pooled effect estimates, P-values, confidence intervals, heterogeneity statistics, meta-regression, and statistical subgroup analyses were not calculated.

## Review

Epidemiological link between psychiatric disorders and cardiometabolic disease

Epidemiological studies consistently show associations among psychiatric disorders, cardiometabolic morbidity, and premature death. A cohort study of severe mental illness (SMI) reported excess mortality with variation across ethnic groups, highlighting the combined influence of psychiatric diagnosis, social disadvantage, inequitable healthcare access, and physical disease burden [16]. This evidence is strongest for schizophrenia, psychotic disorders, and broader SMI populations, whereas cardiometabolic risk (CMR) appears more heterogeneous across depression, anxiety disorders, and bipolar disorder. Evidence grade is as follows: strongly supported for the association between SMI, CMD burden, and premature mortality.

Evidence from the SUN cohort showed that higher cardiovascular risk scores were associated with the later onset of depression, suggesting that vascular and metabolic risk may precede some depressive outcomes rather than only follow psychiatric illness [17]. This supports a probable bidirectional model, including vascular depression and metabolic-inflammation frameworks, but remains vulnerable to confounding by obesity, smoking, inactivity, diet, medication exposure, socioeconomic position, and baseline health status. Evidence grade is as follows: promising but limited for bidirectional vascular-metabolic contributions to depression.

A nationwide cohort study of patients with type 2 diabetes found that comorbid mental disorders were associated with increased cardiovascular disease risk, indicating that psychiatric comorbidity may identify a higher-risk subgroup within established metabolic disease [18]. This supports a reverse-direction pathway in which established metabolic disease may worsen psychiatric vulnerability through vascular burden, inflammatory signalling, treatment complexity, functional limitation, and reduced quality of life. Evidence grade is as follows: strongly supported for higher cardiovascular risk among patients with diabetes and psychiatric comorbidity; promising but limited for specific mechanisms.

An 11-year population-based cohort study reported excess mortality and substantial life-years lost among individuals with schizophrenia and other non-affective psychoses [19]. This mortality signal is well replicated, but interpretation requires attention to antipsychotic exposure, smoking, substance use, poverty, diagnostic delay, fragmented medical care, and the undertreatment of cardiovascular risk factors. Overall, epidemiological evidence is strongest for SMI and psychosis-related mortality, moderate for depression-CMD bidirectionality, and more heterogeneous for anxiety and bipolar disorder; therefore, psychiatric diagnoses should not be treated as having equivalent metabolic risk profiles.

Chronic inflammation as a shared pathway

There is a biologically plausible association between psychiatric disorders and CMD through chronic low-grade inflammation. A population-based two-year longitudinal study found that changes in pro-inflammatory cytokines were associated with late-life depression, suggesting that inflammation may vary over time with depressive symptom burden rather than functioning as a static biomarker [20]. Among proposed biological pathways, inflammation has comparatively strong human evidence because associations have been reported across longitudinal cohorts, meta-analyses, and cross-disorder biomarker studies. However, this evidence is strongest for depression and broader psychiatric biomarker associations, while diagnostic-specific evidence remains less consistent across anxiety, bipolar disorder, and psychotic disorders. Therefore, inflammation should be interpreted as one contributing pathway rather than as a central or disease-specific causal mechanism.

Stress-related neuroinflammation may contribute to the biological plausibility of associations among psychological distress, mood symptoms, and metabolic dysfunction. Mechanistic evidence suggests that inflammatory signalling may influence microglial activation, neurotransmitter metabolism, synaptic plasticity, and HPA axis regulation in major depressive disorder [21]. These processes overlap with cardiometabolic pathways because systemic inflammation has been associated with impaired insulin signalling, endothelial dysfunction, and adipose tissue dysregulation. This supports metabolic-inflammation models in which inflammation may accompany psychiatric illness and may contribute to psychiatric symptoms through vascular, neuroendocrine, and immune pathways. However, much of this evidence remains mechanistic or translational and should not be interpreted as proof of direct causation. The same inflammatory markers are also elevated in obesity, diabetes, infection, ageing, smoking, sleep disturbance, and other medical conditions, limiting their specificity for psychiatric-metabolic comorbidity.

Cross-disorder evidence also suggests that inflammation may act as a transdiagnostic correlate. A cross-disorder assessment of 43 meta-analyses found reproducible inflammation-related biomarker abnormalities across major psychiatric disorders, although some specificity varied by diagnostic category [22]. This supports a shared-pathway model, but inflammatory markers have limited individual-level clinical utility because they are confounded by obesity, ageing, medication exposure, smoking, infection, and medical comorbidities. Thus, inflammatory biomarkers are relatively well replicated at the group level but remain poor stand-alone diagnostic or treatment-selection tools in routine care. In addition, inconsistent assay methods, variable sampling conditions, heterogeneity in psychiatric diagnosis, and differences in illness stage may partly explain why inflammatory findings do not translate reliably into patient-level risk prediction.

Inflammation also overlaps with metabolic and endocrine systems. Evidence that insulin and obesity can alter HPA axis function supports a bidirectional inflammatory-metabolic-endocrine model [23]. The direction of these relationships remains uncertain because inflammation may precede, accompany, or result from psychiatric illness, obesity, medication exposure, and lifestyle risk factors. Overall, inflammation is one of the better-supported biological pathways linking psychiatric and cardiometabolic disorders, but its clinical role is currently stronger for risk conceptualization than for diagnosis, causal attribution, or biomarker-guided treatment. Accordingly, inflammatory biomarkers should be viewed as nonspecific correlates within a broader biopsychosocial and cardiometabolic risk framework, not as validated causal markers or routine clinical decision tools. Key inflammatory and metabolic pathways common to psychiatric illness are summarized in Table 1.

**Table 1:** Inflammatory Mechanisms Linking Psychiatric and Cardiometabolic Disorders. HPA: hypothalamic-pituitary-adrenal

Theme	Main Finding	Study Type	Critical Interpretation	Reference
Inflammation as a dynamic process	Changes in pro-inflammatory cytokines were associated with late-life depression over two years	Population-based longitudinal study	Inflammation may vary with depressive symptom burden rather than acting as a fixed biomarker	[20]
Stress-related neuroinflammation	Inflammatory signalling may affect microglial activation, neurotransmitter metabolism, synaptic plasticity, and HPA axis regulation	Mechanistic/narrative review evidence	These mechanisms provide a biologically plausible framework for how psychological stress may contribute to mood symptoms and metabolic dysfunction, but direct clinical causality remains uncertain	[21]
Inflammation as a transdiagnostic mechanism	Reproducible inflammatory biomarker abnormalities were found across major psychiatric disorders	Cross-disorder meta-analytic synthesis	Inflammatory markers support a transdiagnostic model, but their clinical utility is limited by low specificity and multiple confounders, including obesity, smoking, infection, ageing, medication exposure, and medical comorbidity	[22]
Metabolic-endocrine interaction	Insulin and obesity can alter HPA axis function	Metabolic-endocrine mechanistic study	Metabolic dysfunction may reshape stress biology and amplify inflammatory and neuroendocrine dysregulation	[23]
Overall implication	Narrative synthesis: psychiatric stress, inflammation, obesity, and insulin resistance may interact bidirectionally	Author synthesis of mixed evidence types	Integrated assessment of mood symptoms, inflammatory burden, and metabolic health appears clinically reasonable; however, inflammatory biomarkers should not be interpreted as stand-alone diagnostic or causal markers	[20-23]

Hypothalamic-pituitary-adrenal axis dysregulation and stress biology

Chronic stress may be associated with psychiatric symptoms and CMD through the dysregulation of the HPA axis. Evidence linking racial discrimination and lower education with biological dysregulation suggests that structural stressors may shape endocrine, inflammatory, and metabolic risk [24]. This biological dysregulation may include altered cortisol secretion, flattened diurnal cortisol rhythms, impaired cortisol awakening response, and reduced feedback regulation of the HPA axis. These abnormalities are clinically relevant because cortisol influences glucose metabolism, visceral adiposity, blood pressure, inflammation, sleep regulation, and mood symptoms. This expands the model beyond individual psychiatric vulnerability, but the evidence remains vulnerable to confounding by income, neighborhood exposures, healthcare access, diet, sleep, and baseline medical risk. Social adversity should therefore be interpreted as a contextual risk amplifier rather than a single biological cause of psychiatric-metabolic disease.

There is also an observed association between depression and coronary heart disease (CHD). Review evidence has proposed platelet activation, inflammation, unhealthy behaviors, and HPA axis disturbance as shared mechanisms between depression and CHD [25]. In depression, HPA axis findings may include elevated basal cortisol, altered stress reactivity, and disrupted diurnal cortisol variation, but these patterns are not uniform across all patients or psychiatric diagnoses. This supports a bidirectional model in which depression may worsen cardiovascular risk and cardiovascular illness may worsen depressive symptoms, but it does not establish causality. Among these mechanisms, unhealthy behaviors and reduced preventive care engagement are more clinically actionable than HPA axis biomarkers, which are not routinely used for psychiatric or cardiovascular risk prediction.

Stress-related immune activation further supports this framework. A psychoneuroendocrineimmunology-based review of coronary artery disease described chronic stress, inflammatory signalling, endothelial dysfunction, and neuroendocrine disturbance as interacting pathways [26]. This model is coherent but remains emerging because stress measures, inflammatory markers, and cardiovascular outcomes are not standardized across studies. Longitudinal stress studies are particularly important because they can help determine whether chronic adversity and repeated stress exposure precede endocrine-metabolic dysregulation, rather than merely co-occurring with psychiatric symptoms or CMD. However, longitudinal evidence remains difficult to interpret because cortisol patterns are affected by sleep, medication use, obesity, smoking, comorbid illness, sampling time, and acute stress at measurement.

Psychological traits may also influence risk expression in metabolic disease. Population-based cohort evidence from The Maastricht Study linked psychological and personality factors with variables related to type 2 diabetes, suggesting interaction among affective traits, stress vulnerability, and metabolic dysfunction [27]. Overall, stress biology supports a bidirectional stress-metabolic pathway, but the key clinical implication is the combined assessment of stress exposure, behavioral risk, and conventional CMR markers rather than reliance on HPA axis measures alone. Thus, cortisol abnormalities and diurnal cortisol disruption strengthen the biological plausibility of the stress-CMD link, but they remain insufficiently specific and standardized for routine clinical risk prediction.

Sleep and circadian disturbance

Sleep and circadian disturbance represent clinically relevant pathways through which psychiatric symptoms and cardiometabolic vulnerability may overlap. Insomnia, fragmented sleep, delayed sleep timing, reduced sleep duration, and circadian misalignment are common in depression, anxiety disorders, bipolar disorder, schizophrenia, and SMI. Unlike mitochondrial or microbiome mechanisms, sleep disturbance is directly assessable in routine clinical care and may affect both psychiatric symptoms and metabolic risk through appetite regulation, insulin sensitivity, autonomic tone, inflammatory activity, HPA axis function, physical activity, and dietary behavior.

This relationship appears bidirectional. Poor sleep may worsen fatigue, mood instability, cognitive performance, medication adherence, activity levels, and diet quality, thereby increasing CMR. Conversely, obesity, obstructive sleep apnea, diabetes, chronic pain, and cardiovascular disease may worsen sleep quality and psychiatric symptom burden. However, sleep-related associations are difficult to isolate because they are confounded by medication exposure, illness severity, shift work, socioeconomic stress, obesity, substance use, pain, and sleep-disordered breathing. Current evidence therefore supports the routine assessment of sleep quality, circadian rhythm disruption, and symptoms of sleep-disordered breathing as part of integrated psychiatric and metabolic care. What remains uncertain is whether sleep-targeted interventions independently reduce long-term CMD outcomes in psychiatric populations beyond their effects on mood, activity, diet, and weight.

Insulin resistance, glucose metabolism, and brain function

Insulin resistance and abnormal glucose metabolism are important pathways linking psychiatric disorders with CMD. A systematic review and meta-analysis found abnormal glycemic control in first-episode psychosis, suggesting that some metabolic vulnerability may precede prolonged antipsychotic exposure [28]. This is among the stronger mechanistic-clinical associations because it is supported by human early-illness evidence, although residual confounding by stress, diet, activity, smoking, sleep disruption, substance use, and socioeconomic disadvantage remains possible.

Insulin resistance may also overlap with inflammatory pathways in mood disorders. Neuroinflammation and insulin resistance have been proposed as interacting processes in major depression and bipolar disorder, with possible relevance to future immunometabolic trials [29]. This framework is clinically plausible because insulin signalling affects energy regulation, cognition, reward processing, and neuroplasticity, but immunometabolic treatment strategies remain emerging and are not yet ready for routine biomarker-guided prescribing. Brain insulin signalling is particularly relevant because insulin receptors are expressed in regions involved in cognition, reward processing, appetite regulation, and emotional regulation. Central insulin resistance may therefore provide a mechanistic bridge between peripheral metabolic dysfunction, altered reward sensitivity, cognitive impairment, and mood symptoms.

Glucose dysregulation may also be associated with cognition in schizophrenia. A systematic review and meta-analysis linked glucose homeostasis abnormalities with cognitive domains such as memory, attention, and executive function in schizophrenia [30]. However, directionality remains uncertain because impaired cognition may reduce adherence and self-care, while metabolic dysfunction may affect cognition through vascular, inflammatory, and neuroenergetic mechanisms. This is important because central insulin resistance could plausibly contribute to cognitive dysfunction through impaired neuronal glucose utilization, synaptic plasticity, vascular regulation, and neuroinflammatory signalling. However, direct evidence separating central insulin resistance from peripheral insulin resistance in psychiatric populations remains limited.

**Figure 2:**
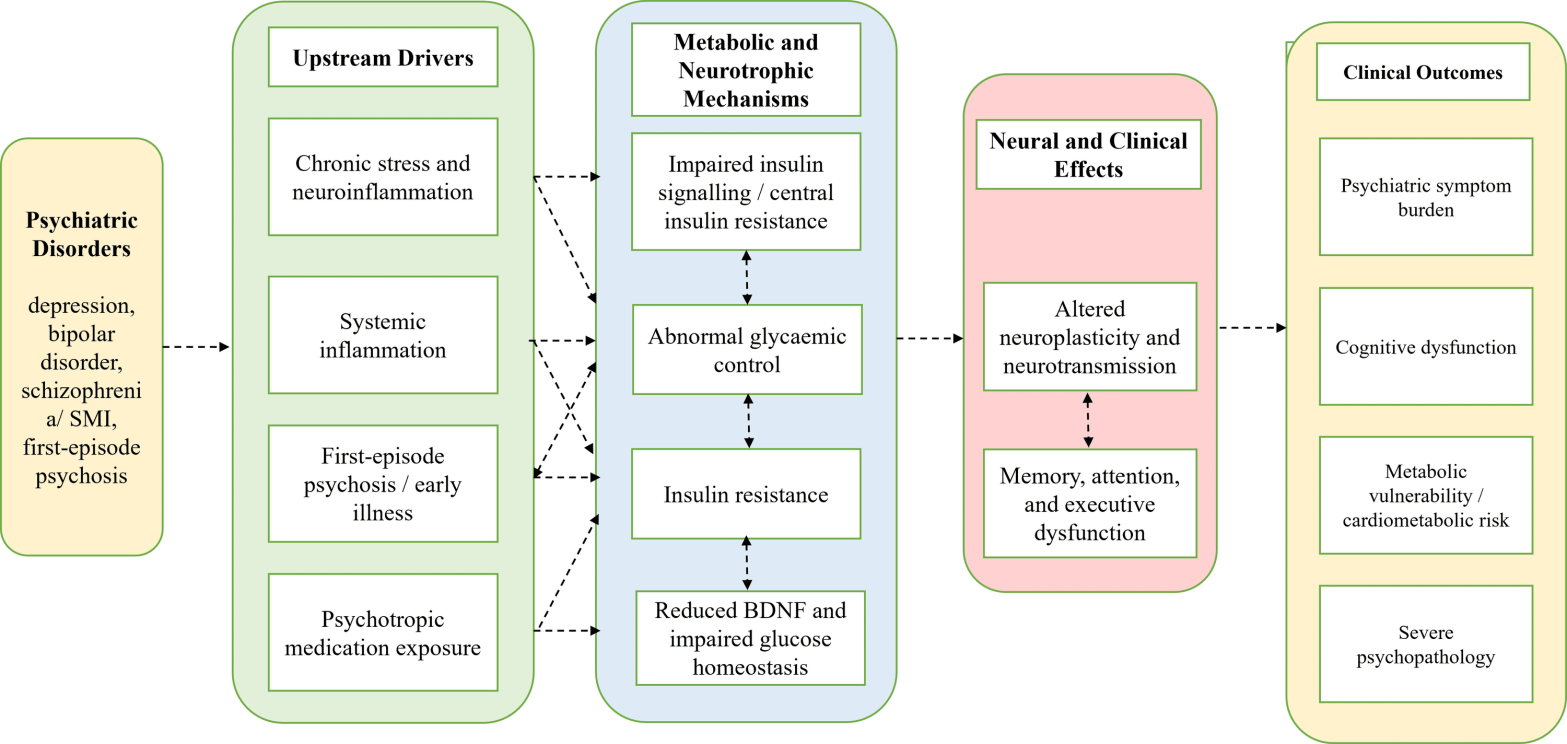
Shared Pathways Linking Psychiatric Disorders, Insulin Resistance, and Cognitive-Metabolic Outcomes. Created by the authors using Microsoft PowerPoint. This schematic illustrates proposed and partially overlapping pathways linking psychiatric disorders with insulin resistance, abnormal glycemic control, neuroinflammation, neurotrophic disruption, cognitive dysfunction, and metabolic vulnerability. Psychotropic medication exposure is included as a clinically important contributor to metabolic risk, although some glucose dysregulation may also be present in early-illness or first-episode psychosis cohorts before prolonged antipsychotic exposure. Dashed arrows indicate proposed associations rather than confirmed causal pathways. SMI, severe mental illness; BDNF, brain-derived neurotrophic factor

Evidence from chronic schizophrenia further suggests associations among systemic inflammation, insulin resistance-related indicators, psychopathology, and brain-derived neurotrophic factor [31]. This links metabolic, inflammatory, and neurotrophic domains but remains biomarker-association evidence rather than proof of a clinically actionable causal pathway. Emerging insulin-sensitizing approaches, including metformin and other metabolic interventions, are relevant to psychiatric-metabolic care, particularly for antipsychotic-associated weight gain and insulin resistance. However, these strategies are better supported for cardiometabolic risk reduction than for the direct improvement of core psychiatric symptoms, and evidence for newer insulin-sensitizing or immunometabolic therapies remains investigational. The strongest clinical implication is routine screening with established measures such as fasting glucose, HbA1c, lipids, blood pressure, body mass index (BMI), and waist circumference, rather than reliance on experimental neuroinflammatory or neurotrophic biomarkers. Figure 2 summarizes how insulin resistance, systemic inflammation, altered glucose metabolism, central insulin signalling abnormalities, and reduced neurotrophic signalling may be associated with psychiatric symptom burden, cognitive dysfunction, and metabolic vulnerability.

Oxidative stress, mitochondrial dysfunction, and energy metabolism

Oxidative stress and mitochondrial dysfunction are biologically plausible mechanisms associated with psychiatric illness and CMD. A review of mitochondrial dysfunction in psychiatric disorders described reduced energy production, altered calcium signalling, increased reactive oxygen species, and disrupted neuronal communication [32]. These abnormalities may be relevant to mood regulation, cognition, stress tolerance, and neuroplasticity because the brain has a high metabolic demand. However, this pathway remains emerging rather than clinically established because current evidence is largely correlative and does not clarify whether mitochondrial dysfunction is a cause, consequence, or marker of psychiatric illness.

Energy metabolism has also been discussed in relation to mood disorders. A narrative review of mood disorders described the mitochondrial hypothesis and proposed potential metabolic biomarkers for future investigation [33]. Symptoms such as fatigue, anhedonia, cognitive slowing, and treatment resistance may be associated with impaired cellular energetics, but these biomarkers lack validated thresholds, reproducibility across clinical populations, and routine diagnostic utility.

Oxidative stress-induced mitochondrial injury is also implicated in diabetic microvascular complications, with potential effects on vascular, renal, retinal, neural, and brain systems [34]. This supports a shared systemic bioenergetic framework linking peripheral metabolic disease with possible neuropsychiatric vulnerability, but findings from diabetes cannot be directly extrapolated to psychiatric populations because medication exposure, lifestyle risk, illness chronicity, and baseline CMR differ substantially.

Brain metabolic changes have also been related to cognitive dysfunction and neuropsychiatric symptoms [35]. Overall, oxidative stress, mitochondrial dysfunction, inflammation, vascular injury, and impaired neural resilience may represent overlapping biological processes rather than a single causal pathway. The strongest conclusion is that oxidative and mitochondrial pathways are biologically plausible and relevant for future research; the weakest point is clinical translation, because no mitochondrial biomarker is currently validated for routine psychiatric-metabolic screening, prognosis, or treatment selection. Bioenergetic mechanisms of psychiatric symptoms and cardiometabolic dysfunction are summarized in Table 2.

**Table 2:** Oxidative and Mitochondrial Pathways in Psychiatric-Metabolic Comorbidity. Reactive oxygen species (ROS) refers to chemically reactive oxygen-containing molecules that can contribute to oxidative cellular injury when antioxidant defenses are insufficient. Bioenergetics refers to cellular energy production and utilization, particularly mitochondrial ATP generation. Mitochondrial dysfunction refers here to impaired mitochondrial energy production, altered calcium handling, increased oxidative stress, or disrupted cellular signalling relevant to brain and metabolic function. ATP, adenosine triphosphate; CMD, cardiometabolic disease

Theme	Main Finding	Critical Interpretation	Reference
Mitochondrial impairment in psychiatric disorders	Mitochondrial dysfunction may involve reduced energy production, altered calcium signalling, increased reactive oxygen species (ROS), and disrupted neuronal communication	These abnormalities may affect mood regulation, cognition, stress tolerance, and neuroplasticity, but current evidence remains largely associative	[32]
Energy metabolism in mood disorders	The mitochondrial hypothesis links mood symptoms with impaired cellular energetics and potential metabolic biomarkers	Fatigue, anhedonia, cognitive slowing, and treatment resistance may reflect energy deficits; however, this remains a developing framework, and current biomarkers are not validated for routine diagnostic use	[33]
Oxidative injury in metabolic disease	Oxidative stress-induced mitochondrial injury contributes to diabetic microvascular complications	This supports a shared systemic bioenergetic pathway that may affect both peripheral metabolic organs and the brain, linking metabolic disease mechanisms with possible neuropsychiatric vulnerability	[34]
Brain metabolism and neuropsychiatric symptoms	Altered brain metabolism may contribute to cognitive impairment and neuropsychiatric symptoms	Disrupted metabolic signalling may reduce neural resilience and worsen psychiatric morbidity, although clinical translation remains limited by biomarker variability and the lack of validated thresholds	[35]
Overall implication	Narrative synthesis: oxidative stress, mitochondrial dysfunction, inflammation, vascular injury, and impaired neural resilience may jointly link psychiatric morbidity and CMD	Oxidative and mitochondrial mechanisms are biologically plausible contributors to psychiatric-metabolic comorbidity, but current evidence is predominantly associative and lacks sufficient longitudinal and interventional confirmation	[32-35]

Gut-brain-metabolic axis and microbiome alterations

The gut-brain-metabolic axis provides a biologically plausible pathway through which psychiatric disorders may be associated with metabolic dysfunction via microbiome-mediated effects on immunity, neurotransmission, and host energy regulation. Metagenome-wide evidence has identified gut microbiome features associated with schizophrenia, suggesting altered microbial composition or functional capacity in psychotic disorders [36]. However, microbiome evidence remains exploratory compared to inflammation or mortality data because microbial profiles are highly sensitive to diet, geography, antibiotic exposure, psychotropic medication, gastrointestinal comorbidity, body mass index, illness chronicity, and sequencing methodology.

Microbiome associations also extend to mood symptoms. A large microbiome-wide association study found links between gut microbial profiles and depressive symptoms, but the effect sizes were modest, and population variability limits immediate clinical translation [37]. Thus, the microbiome is better interpreted as an emerging correlate of psychiatric-metabolic vulnerability than as an established diagnostic or therapeutic target. At present, no gut microbiome signature is sufficiently validated to guide routine psychiatric diagnosis, CMD risk prediction, or individualized treatment selection.

Microbiome pathways may also be involved in treatment-related metabolic risk. A systematic review reported associations between gut microbiome alterations, antipsychotic-induced weight gain, and metabolic dysfunction [38]. This area is highly confounded because antipsychotic exposure, appetite changes, weight gain, diet composition, constipation, reduced physical activity, and illness severity may all alter microbiome composition. Therefore, microbiome changes during antipsychotic treatment should not be assumed to mediate metabolic adverse effects without longitudinal and interventional confirmation.

Associations between gut microbiome alterations, metabolic changes, and metabolic syndrome severity have also been reported in schizophrenia [39]. Overall, the findings support a possible bidirectional gut-brain-metabolic model, but randomized microbiome interventions, standardized diet and medication data, and longitudinal studies are still required before microbiome-based care can be used routinely in psychiatry. The strongest current conclusion is that microbiome findings generate mechanistic hypotheses about psychiatric-metabolic overlap; the weakest point is clinical utility, because evidence remains heterogeneous, effect sizes are modest, and interventional data are insufficient.

Psychotropic medications and treatment-related metabolic risk

Psychotropic medications, particularly antipsychotics, are an important treatment-related contributor to cardiometabolic risk in psychiatric populations. Antipsychotics are clinically necessary for many patients with schizophrenia and other psychotic disorders because they reduce relapse risk, improve symptom control, and support long-term psychiatric stability. However, antipsychotic agents differ substantially in their associations with weight gain, insulin resistance, dyslipidemia, glucose abnormalities, and broader CMR. A systematic review and network meta-analysis comparing 18 antipsychotics found clinically relevant differences in metabolic effects across individual drugs, supporting the need for individualized prescribing rather than treating antipsychotics as a metabolically uniform class [40].

Medication choice should therefore balance psychiatric efficacy, relapse prevention, adherence, prior treatment response, tolerability, and baseline cardiometabolic vulnerability. Evidence comparing oral and long-acting injectable antipsychotics for schizophrenia maintenance treatment further supports individualized decision-making, because treatment effectiveness and tolerability vary across agents and formulations [41]. In patients with preexisting obesity, diabetes, dyslipidemia, hypertension, or a strong family history of CMD, baseline CMR should be considered before antipsychotic initiation or switching. Treatment decisions should also account for illness severity and relapse risk, because undertreated psychosis may itself worsen physical health through poor self-care, inactivity, substance use, disrupted sleep, and fragmented healthcare engagement.

Weight gain is one of the most visible antipsychotic-related adverse effects, but it should not be used as the only marker of metabolic harm. A meta-analysis of specific antipsychotic drugs found variable weight-gain risk across agents [42]. However, antipsychotic-associated metabolic dysfunction may also involve central adiposity, insulin resistance, HbA1c elevation, triglyceride increases, high-density lipoprotein (HDL) reduction, and blood pressure changes. Therefore, monitoring should include weight/BMI, waist circumference, blood pressure, fasting glucose or HbA1c, and lipid profile at baseline and during follow-up, particularly after antipsychotic initiation or medication change.

Preventive metabolic management is also relevant. Consensus guidance supports considering metformin in selected patients at risk of antipsychotic-induced weight gain, particularly when lifestyle measures alone are insufficient or when higher-risk antipsychotics are clinically required [43]. Metformin should be considered alongside lifestyle counselling, shared decision-making, and structured metabolic monitoring rather than as a universal intervention for all patients. Because antipsychotic agents differ in their associations with weight gain, glucose-lipid abnormalities, and broader CMR, treatment decisions should balance psychiatric efficacy with baseline metabolic vulnerability and ongoing monitoring, as summarized in Table 3.

**Table 3:** Treatment-Related Metabolic Risks of Antipsychotic Medications. Suggested cardiometabolic monitoring parameters include baseline and follow-up weight/BMI, waist circumference, blood pressure, fasting glucose or HbA1c, and lipid profile, with closer monitoring after antipsychotic initiation or medication change. BMI, body mass index; CMR, cardiometabolic risk; HDL, high-density lipoprotein

Theme	Main Finding	Critical Interpretation	Reference
Drug-specific metabolic effects	Antipsychotics differ substantially in their effects on weight, glucose metabolism, and lipid profiles	Metabolic risk should be considered drug-specific, supporting individualized prescribing for patients with cardiometabolic vulnerability	[40]
Balancing efficacy and metabolic safety	Oral and long-acting injectable antipsychotics differ in efficacy and tolerability for schizophrenia maintenance treatment	Medication choice should balance relapse prevention, adherence, prior response, tolerability, and physical health risk, including the patient’s baseline CMR	[41]
Antipsychotic-associated weight gain	Specific antipsychotics carry different risks of weight gain	Weight gain is an early warning sign, but it does not fully capture insulin resistance, dyslipidemia, hypertension, central adiposity, or longer-term cardiovascular risk	[42]
Broader metabolic monitoring	Antipsychotic-related metabolic risk may involve glucose dysregulation, HbA1c elevation, triglyceride and HDL changes, increased waist circumference, and blood pressure elevation	Monitoring should extend beyond weight alone and include standard cardiometabolic measures that are clinically actionable and comparable over time	[40-42]
Preventive metabolic management	Consensus guidance supports considering metformin in selected patients at risk of antipsychotic-induced weight gain	Metformin should be considered alongside lifestyle counselling, shared decision-making, and structured metabolic monitoring rather than as a universal intervention for all patients	[43]
Overall implication	Narrative synthesis: antipsychotic treatment can be clinically necessary but requires structured cardiometabolic risk management	Shared decision-making, baseline assessment, and regular monitoring are essential to reduce treatment-related cardiometabolic harm	[40-43]

Clinical implications: Screening, prevention, and integrated care

Regular psychiatric care should include cardiometabolic prevention. Physical activity has been associated with a reduced risk of depression and anxiety, suggesting dual relevance for psychiatric symptoms and metabolic health [44]. Exercise may improve mood, insulin sensitivity, weight regulation, blood pressure, and inflammation, but implementation is limited by negative symptoms, fatigue, medication sedation, socioeconomic barriers, stigma, and limited service access. Among proposed interventions, physical activity and structured lifestyle support have stronger clinical relevance than most biomarker-based approaches because they are modifiable, low cost, and linked to both mental and cardiometabolic outcomes.

Lifestyle intervention is particularly important during antipsychotic treatment. Evidence supports lifestyle intervention for preventing or controlling antipsychotic-induced weight gain in SMI [45]. This area has more direct interventional support than many mechanistic pathways discussed in this review, but benefits depend on intervention intensity, timing, adherence support, follow-up, and integration with psychiatric prescribing. Lifestyle intervention should therefore be presented as evidence-supported but implementation-sensitive, not as a uniformly effective solution.

Scalable models are also needed. The LION cluster randomized controlled trial evaluated a multimodal web-based lifestyle intervention for cardiometabolic health in serious mental illness [46]. Digital approaches may extend access, but engagement, digital literacy, clinician support, cognitive impairment, internet access, literacy, and housing instability influence effectiveness. Randomized trial evidence gives digital and multimodal lifestyle models greater evidentiary weight than consensus-only recommendations, but digital delivery may widen inequities if access barriers are not addressed.

Pooled randomized trial evidence also supports weight-management and health-promotion interventions in psychosis, although certainty is limited by variation in intervention design, study quality, and follow-up duration [47]. Psychiatric services should routinely monitor BMI, waist circumference, blood pressure, fasting glucose or HbA1c, and lipid profile and coordinate care with primary care, endocrinology, cardiology, dietetics, and behavioral support services. The most established clinical recommendation is routine monitoring and the management of conventional CMR factors because these measures are validated, inexpensive, and actionable. By contrast, inflammatory, neurotrophic, mitochondrial, and microbiome biomarkers remain insufficiently validated for routine clinical decision-making. The practical priority is integrated care combining standard metabolic screening, medication-risk review, smoking cessation, sleep assessment, diet and activity support, and timely referral for diabetes, dyslipidemia, hypertension, and cardiovascular disease management.

Limitations and future directions

There are a few limitations to this review. First, although a narrative methodology has been added to improve transparency, this review was not designed as a systematic review or meta-analysis; therefore, publication bias, selective citation, and the incomplete retrieval of relevant studies remain possible. There is heterogeneity of the evidence in terms of psychiatric diagnosis, cardiometabolic outcomes, definitions of biomarkers, study design, and follow-up duration. Diagnostic variability across psychiatric disorders also limits direct comparison among studies, because depression, anxiety disorders, bipolar disorder, schizophrenia, and broader SMI categories may differ substantially in illness course, medication exposure, behavioral risk, and healthcare access. Some of the studies are observational or review studies and thus do not allow for causal inferences. As a result, most associations described in this review should be interpreted as hypothesis-supporting rather than causal. The confounding factors, such as exposure to psychotropic medications, the severity of illness, smoking history, dietary habits, the lack of physical activity, socioeconomic status, and preexisting medical disease, may affect the observed relationship. Moreover, there are shared mechanisms, such as inflammation, HPA axis dysfunction, mitochondrial dysfunction, and changes in the gut microbiome, that are biologically connected and are hard to disentangle from one another. Biomarker interpretation is further limited by heterogeneity in measurement methods, assay platforms, sampling time, clinical state, medication exposure, infection status, obesity, and comorbid medical disease. This limitation is central to the interpretation of the review: epidemiological links between SMI, CMD, and mortality are relatively well established, whereas several mechanistic pathways, including mitochondrial dysfunction and microbiome alteration, remain more speculative and less clinically validated.

A key unresolved issue is the extent to which metabolic dysfunction reflects primary disease biology, treatment exposure, chronic illness burden, social adversity, behavioral risk, or an interaction among these domains. Evidence from first-episode psychosis and early-illness populations suggests that some glucose dysregulation and metabolic vulnerability may be present before prolonged antipsychotic exposure, supporting the possibility of illness-associated metabolic risk. In chronic psychiatric illness, however, cumulative effects of long-term medication exposure, recurrent symptoms, reduced activity, poor diet quality, smoking, sleep disturbance, poverty, and fragmented healthcare may become difficult to separate from primary disease effects. Antipsychotic exposure is an especially important treatment-related contributor because specific agents differ in their associations with weight gain, glucose-lipid abnormalities, and broader CMR. Therefore, metabolic dysfunction in psychiatric populations is best interpreted as a layered phenomenon involving early illness-related vulnerability, chronic disease burden, treatment-related metabolic effects, and social-behavioral confounding rather than as a single pathway. Accordingly, evidence should be interpreted across four overlapping domains: medication-naïve or early-illness findings, chronic psychiatric disease burden, cumulative psychotropic exposure, and social-behavioral determinants. This distinction is necessary because the same cardiometabolic phenotype may arise from different combinations of illness biology, treatment effects, lifestyle risk, and structural disadvantage.

The evidence base also varies substantially by domain. Conventional CMR measures, including body mass index, waist circumference, blood pressure, fasting glucose or HbA1c, and lipid profile, have the strongest clinical utility because they are standardized, reproducible, and linked to actionable interventions. In contrast, inflammatory, neurotrophic, mitochondrial, and microbiome biomarkers may show replication at the group level but currently have limited individual-level diagnostic or prognostic value. Similarly, lifestyle and weight-management interventions have some randomized evidence, whereas many immunometabolic, microbiome-directed, and biomarker-guided strategies remain investigational. Emerging precision psychiatry approaches, including pharmacogenomics and biomarker-guided treatment selection, were not reviewed in depth and remain important areas for future psychiatric-metabolic research.

Future research should focus on longitudinal cohorts and randomized controlled trials to determine the causation and direction of the relationship between psychiatric illness and CMD. There is a need for the standardization of the assessment of metabolic indices, inflammatory biomarkers, microbiome profile, lifestyle, and medication exposure to enhance comparability among studies. Integrated approaches to care, early metabolic screening approaches, tailored psychotropic treatments, lifestyle interventions, and immunometabolic therapies should also be explored in future studies. Diversity will be important for the improvement of generalizability and for the identification of setting-specific risk factors. Future studies should also explicitly model major confounders, including obesity, smoking history, sedentary behavior, diet quality, sleep disturbance, socioeconomic disadvantage, illness severity, and psychotropic exposure. Study designs should stratify participants by medication-naïve or early-illness status, chronic psychiatric disease burden, antipsychotic class and the duration of exposure, and social-behavioral risk profiles. This separation would help clarify which metabolic abnormalities are present early in the illness course, which emerge with chronicity, which are treatment-associated, and which are primarily explained by modifiable behavioral or structural determinants. The greater inclusion of underrepresented populations and low-resource settings is needed to determine whether psychiatric-metabolic associations differ by ethnicity, sex, age, geography, healthcare access, and social adversity.

## Conclusions

This review shows that psychiatric disorders and CMD are associated through overlapping inflammatory, neuroendocrine, metabolic, mitochondrial, gut-brain, sleep-related, behavioral, and treatment-related pathways. Current evidence supports a strong association and probable bidirectional relationship between psychiatric and cardiometabolic disorders, particularly in SMI, schizophrenia, psychotic disorders, and depression. Given the breadth of this topic, the review should be interpreted as a clinically oriented narrative synthesis rather than a comprehensive disorder-by-disorder evidence map. The strongest and most consistent evidence concerns SMI, particularly schizophrenia and psychotic disorders, where excess CMD burden, antipsychotic-related metabolic risk, and premature mortality are well replicated; evidence for depression is also substantial, especially for inflammation, vascular risk, diabetes comorbidity, and bidirectional cardiometabolic associations. By contrast, anxiety, bipolar disorder, sleep/circadian disturbance, mitochondrial dysfunction, microbiome alteration, neurotrophic markers, and biomarker-guided pathways remain comparatively emerging or less fully developed.

Cardiometabolic dysfunction should therefore be regarded as an important component of psychiatric morbidity rather than only a secondary comorbidity. Treatment should focus on psychiatric symptom control while also including metabolic screening, individualized psychotropic prescribing, lifestyle modification, sleep assessment, early prevention, and multidisciplinary care involving psychiatry, primary care, endocrinology, cardiology, nutrition, and behavioral health services. These recommendations are strongest where they rely on established CMR parameters and antipsychotic monitoring practices and more tentative where they involve immunometabolic, mitochondrial, microbiome-directed, or biomarker-guided interventions. Many proposed mechanistic pathways remain incompletely understood and require further longitudinal and interventional investigation to clarify causality, directionality, and clinical utility. Longitudinal and interventional studies are warranted to clarify causal pathways, distinguish primary illness effects from treatment-related and social-behavioral contributors, and evaluate integrated models that improve both psychiatric and cardiometabolic outcomes.
